# Cost-effectiveness analysis of anastrozole *vs* tamoxifen in adjuvant therapy for early stage breast cancer in the United Kingdom: the 5-year completed treatment analysis of the ATAC (‘Arimidex’, Tamoxifen alone or in combination) trial

**DOI:** 10.1038/sj.bjc.6603804

**Published:** 2007-07-10

**Authors:** R Mansel, G Locker, L Fallowfield, Á Benedict, D Jones

**Affiliations:** 1 Department of Surgery, Wales College of Medicine, Cardiff University, Heath Park, Cardiff, CF14 4XN, UK; 2 Department of Oncology, Evanston Northwestern Healthcare, Evanston, IL, USA; 3 Department of Psycho-oncology, Brighton & Sussex Medical School, Brighton, BN1 9QG, UK; 4 MEDTAP International Inc., 20 Bloomsbury Square, London, WC1A 2NS, UK; 5 AstraZeneca, Parklands, Macclesfield, SK10 4TF, UK

**Keywords:** anastrozole, tamoxifen, aromatase inhibitor, breast cancer, cost-effectiveness analysis, cost-utility analysis

## Abstract

Results from the completed treatment analysis of the ATAC (Arimidex, Tamoxifen alone or in combination) trial indicated that anastrozole was significantly superior to tamoxifen in terms of efficacy and safety in the adjuvant treatment of postmenopausal women with hormone receptor-positive (HR+) early breast cancer. On the basis of these results, this study estimated the cost-effectiveness of anastrozole *vs* tamoxifen, from the perspective of the UK National Health Service (NHS). A Markov model was developed using the 5-year completed treatment analysis from the ATAC trial (ISRCTN18233230), as well as data obtained from published literature and expert opinion. Resource utilisation data and associated costs (2003–4 UK£) were compiled from standard sources and expert opinion. Utility scores for a number of health states were obtained from a cross-sectional study of 26 representative patients using the standard gamble technique. The utility scores were then inserted into the model to obtain cost per quality adjusted life-year (QALY) gained. Costs and benefits were discounted at recommended annual rates of the UK Treasury (3.5%). Modelled for 25 years, anastrozole, relative to generic tamoxifen, was estimated to result in 0.244 QALYs gained per patient at an additional cost of £4315 per patient). The estimated incremental cost-effectiveness of anastrozole compared with tamoxifen was £17 656 per QALY gained. There was a greater than 90% probability that the cost-effectiveness of anastrozole was below £30 000 per QALY gained and of the order of 65% that it was below £20 000 per QALY gained. The results were robust to all parameters tested in sensitivity analysis. Compared with commonly accepted thresholds, anastrozole is a cost-effective alternative to generic tamoxifen in adjuvant treatment of postmenopausal women with HR+ early breast cancer from the UK NHS perspective.

Breast cancer is common among women in the United Kingdom, causing the death of around 70 women per 100 000 aged 50–69 years on an annual basis (Peto *et al*, 2000). Treatment of this common malignancy is a significant economic burden. For example, the annual cost to the UK National Health Service (NHS) of drug therapy for 13 200 patients with hormone receptor-positive (HR+) early breast cancer has been estimated at £64.6 million (Benedict and Christie, 2003). Tamoxifen is an established adjuvant treatment option in this regard (Early Breast Cancer Trialists' Collaborative Group, 1998; Early Breast Cancer Trialists' Collaborative Group, 2005). However, treatment with tamoxifen has been associated with serious safety concerns (Reddy and Chow, 2000), including thromboembolic disorders (Love *et al*, 1991) and increased risk of the development of endometrial cancer (Fisher *et al*, 2001). Consequently, there is a need for new hormonal treatment options with improved safety and efficacy, particularly in terms of avoiding or delaying costly disease recurrences (Lamerato *et al*, 2004).

Anastrozole is an aromatase inhibitor that has proved significantly superior to tamoxifen as adjuvant treatment for postmenopausal women with HR+ early breast cancer. This was apparent in terms of more favourable disease-free survival (hazard ratio 0.83, *P*=0.005) and longer time to recurrence (Hazard Ratio 0.74, *P*=0.0002) in the completed treatment analysis of the ATAC (Arimidex, Tamoxifen Alone or in Combination) trial (ISRCTN18233230), which was performed in postmenopausal women with early operable breast cancer who had completed primary therapy and were eligible for adjuvant hormonal therapy (ATAC Trialists' Group, 2005). Anastrozole was also associated with a more favourable safety profile *vs* tamoxifen, including a significantly lower incidence of endometrial cancer, thromboembolic events and vaginal bleeding/discharge albeit with increased risk of arthralgia and bone fracture (ATAC Trialists' Group, 2005). These results confirmed the previously reported findings at a median 47 months' follow-up (The ATAC Trialists' Group, 2003). Furthermore, in a subprotocol of the ATAC trial, it was shown that the superiority of anastrozole over tamoxifen was achieved without a detrimental impact on overall quality of life (Fallowfield *et al*, 2004; Cella *et al*, 2006).

The findings of the ATAC trial demonstrate that anastrozole (1 mg daily) provides an effective and well-tolerated alternative to tamoxifen (20 mg daily) for adjuvant treatment of postmenopausal women with HR+ early breast cancer. Subsequently, the ASCO Technology Assessment recommended that optimal adjuvant hormonal therapy for a postmenopausal woman with HR+ early breast cancer should now include an aromatase inhibitor either as initial therapy or after treatment with tamoxifen (Winer *et al*, 2005). However, the higher acquisition cost of anastrozole, against a background of increasing fiscal constraints on healthcare budgets, could limit the adoption of this agent as has occurred for other new therapeutic interventions in the United Kingdom and elsewhere. Consequently, healthcare decision makers such as the National Institute for Health and Clinical Excellence (NICE) and the Scottish Medicines Consortium (SMC) recognise that value for money should be a key criterion for deciding which new healthcare interventions should be reimbursed from the limited NHS budget. Evidence from cost-effectiveness analysis, preferably over the expected lifetime of the patient population, is formally used as part of the technology appraisal and subsequent guidance issued to the NHS. On the basis of the evidence submitted, anastrozole has now been recommended by both NICE and the SMC as an option for the adjuvant treatment of early oestrogen-receptor positive invasive breast cancer in postmenopausal women. On the basis of discount rates that were applicable prior to 2004 (6% for costs, 1.5% for benefits), NICE estimated that the cost per quality-adjusted life years (QALY) of anastrozole compared with tamoxifen (£12 310/QALY) lay below the currently accepted thresholds under which interventions are considered to be cost-effective (£20K to £30K/QALY). This study uses the latest recommended discount rates of 3.5% for both costs and benefits and is therefore in accordance with the latest guidelines from NICE (National Institute for Health and Clinical Excellence, 2004).

## MATERIALS AND METHODS

In the ATAC trial, postmenopausal women with early (invasive, operable) breast cancer who had completed primary therapy (surgery±radiotherapy±chemotherapy) and who were eligible for adjuvant hormonal therapy were randomised to receive either anastrozole or tamoxifen (or both) for up to 5 years (ATAC Trialists' Group, 2005). Since the combination arm demonstrated no benefit over tamoxifen in terms of efficacy or safety, this arm was discontinued and the present analysis is restricted to the two monotherapy arms.

### Design of the Markov model, inputs and assumptions

**Figure 1:**
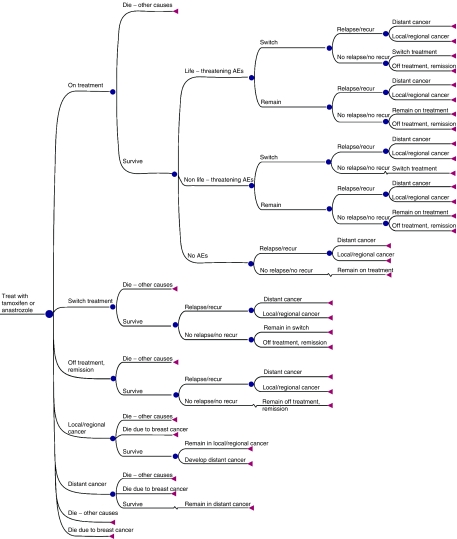
The Markov decision model for health status. AEs, adverse events.

On the basis of the latest results from the ATAC trial, as well as data derived from interviews with UK oncologists and published literature, a probabilistic Markov model (Sonnenberg and Beck, 1993) was developed to project outcomes for a hypothetical cohort of 1000 postmenopausal women (mean age 64 years) with HR+ early breast cancer in the United Kingdom. Markov models assume that a patient is always in one of a finite number of discrete health states and all events are represented as transitions from one state to another. In this study, the Markov model was evaluated as a Monte Carlo simulation. The model projected outcomes over an actuarial time horizon of 25 years. At any time point, women in the model are in one of seven health states: (1) on adjuvant endocrine treatment (i.e. anastrozole or tamoxifen); (2) on an unplanned switch of adjuvant treatment; (3) off-treatment, in remission; (4) with distant recurrence; (5) with local/regional recurrence; (6) dead due to breast cancer or (7) dead due to other causes. All women enter the model in the ‘on adjuvant treatment’ health state (Figure 1). Women would then move between the health states at 3-month intervals during the first 5 years (when they received primary adjuvant treatment with anastrozole or tamoxifen) and at 6-month intervals thereafter (Figure 1).

**Figure 2:**
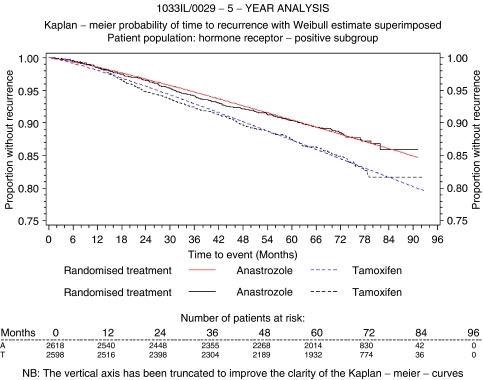
Weibull model fitted to each treatment arm of ATAC.

Event rates for recurrence and breast cancer-related death were derived from the ATAC trial and extrapolated to 25 years using Weibull parametric methods (Cook *et al*, 1969). This involves postulating a Weibull distribution for time to recurrence and estimating the parameters of the distribution from the data. In this study, a Weibull curve was fitted to the time to recurrence data for each treatment arm of ATAC. The Weibull regressions were produced using the LIFEREG procedure of the SAS statistical package. The intercept was estimated as 9.17, the scale parameters as 0.83 and the coefficient of the treatment effect as 0.25. The curve provided a good fit to the data as shown in Figure 2. The probability of recurrence, based on the Weibull curves, was then extrapolated to 10 years from initiation of treatment based on an assumption that the benefit of anastrozole would carry over to 5 years following the end of treatment. This key assumption was based on the continuing divergence of the recurrence curves observed in the completed treatment analysis of the ATAC trial (median 68 months' follow-up) (ATAC Trialists' Group 2005) and the durable treatment benefit previously observed with tamoxifen (Early Breast Cancer Trialists' Collaborative Group, 2005).

**Figure 3:**
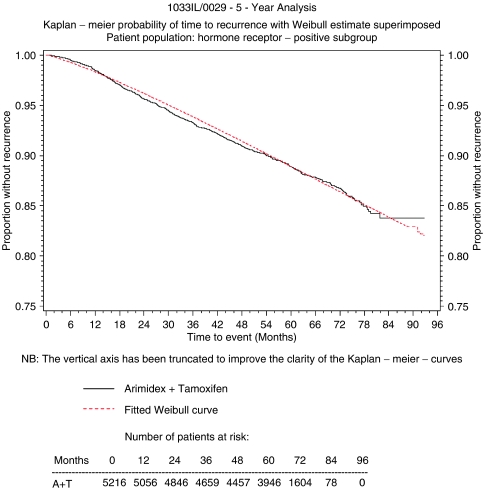
Weibull model fitted to the pooled ATAC data.

A Weibull curve was also fitted to the ATAC time to recurrence data, pooled across the treatment arms. Using the LIFEREG procedure of the SAS statistical package, the intercept was estimated as 9.29 and the scale parameter as 0.83. The curve provided a good fit to the data as shown in Figure 3. The pooled curve was used to calculate probability of recurrence from 10 to 25 years from initiation of treatment based on an assumption that the time-dependent rates of recurrence after 10 years would be the same for both anastrozole and tamoxifen. The Weibull curves provided a good fit to the ATAC trial data and formed the basis for estimating the probability of a patient moving to the recurrence health state, at each cycle in the Markov model, over a 25-year time horizon. The recurrent patients in the model had either a local-regional recurrence or distant recurrence based on the proportion of the two types in the ATAC trial. Progression of patients from recurrence was modelled parsimoniously (see Figure 1). Patients with local-regional recurrence could stay in that state, progress to distant metastasis, or die, either of breast cancer or for other causes. Distant metastatic patients could survive, or die. A 25-year time horizon was chosen for the model because of the need to capture the long-term benefits of treatment. At the same time due to average age at diagnosis and the life expectancy of postmenopausal women with early breast cancer 25 years represents a time period over which the majority of patients would have died. A longer time horizon would have had very little impact on the results. In the basecase analysis it was assumed that the recurrence curves continued to show incremental benefit for anastrozole out to 10 years from initiation of treatment.

The probabilities of other variables used in the model (e.g. adverse events, withdrawal and switching rates) were derived from the 5-year results of the ATAC trial (ATAC Trialists' Group, 2005), other published sources, and an expert panel consisting of six practising UK breast cancer specialists. Other outcome probabilities used in the model for the probabilistic sensitivity analysis, with specified distributions and the sources are shown in Table 1. Adverse events were assumed only to occur during the 5-year treatment period, except for a few events that, in the opinion of the expert panel, patients could remain at risk of beyond treatment completion. In particular, based on the same expert opinion, the model assumed that the risk of hip fracture and endometrial cancer extended for an additional 3 years and that the risk of thromboembolic events extended for an additional 6 months. Hysterectomy rates obtained from the ATAC trial (5.1% with tamoxifen and 1.3% with anastrozole), together with their associated costs and utilities, were also included in the model. To reflect routine clinical practice in the United Kingdom, the cost of endometrial cancer monitoring in patients receiving tamoxifen was excluded and the cost of adding bisphosphonate therapy in anastrozole-treated patients was included. It was assumed that 5% of patients received bisphosphonates during anastrozole treatment, based on bisphosphonate usage recorded in the ATAC trial and supported by expert opinion. The uncertainty in the above assumptions was accounted for in the sensitivity analysis.

Age-specific mortality data for women in the general population were obtained from the UK Office of National Statistics (2002). It was assumed that the risk of death for causes other than breast cancer (including those related to adverse events) was the same for both treatment groups. Mortality statistics from potentially fatal adverse events (e.g. endometrial cancer, thromboembolic events, hip fractures) were also retrieved from the UK Office of National Statistics database and incorporated into the model. It was assumed that mortality due to hip fractures increased with increasing age while death due to other adverse events remained constant over time.

### Resource utilisation and costs

Data on resource utilisation were obtained from structured interviews with the expert panel and, where possible, published literature. This included data on: the medical management required during treatment and follow-up and when patients were off-treatment (due to remission or adverse events); the medical management required at diagnosis and during disease recurrence (local/regional and distant); the treatment of serious adverse events requiring hospitalisation and non-serious adverse events; and palliative care requirements (Table 2). Costs were then assigned to each unit of resource to estimate total costs. Unit cost information was obtained from MEDTAP's unit cost database and published sources, the British National Formulary (2003/2004) and NHS reference costs (Department of Health, 2003). The model only included direct medical costs.

Drug costs were derived from standard sources (anastrozole, £68.56 per 28-tablet pack (MIMS, 2005); tamoxifen, £2.24 per 30-tablet pack (British National Formulary, 2003/2004)). These were not varied in the model. Future costs and benefits were discounted at the annual rate of 3.5%, the rate recommended by the UK Treasury (National Institute for Health and Clinical Excellence).

### Calculating quality-adjusted life years

A cross-sectional study of 26 representative UK patients with early or advanced breast cancer (mean age 68 years) was used to derive quality-of-life weights (utilities) for insertion into the model. Most patients had HR+, node-negative disease and were presently receiving tamoxifen; a minor proportion was receiving anastrozole (no patient was receiving treatment within the ATAC trial). Utilities for different health states were elicited using a chained standard gamble method that compared the health states to perfect and worse health and then worse health against perfect health and death (Torrance, 1986). Table 3 summarises the mean utility scores for the 14 health states plus the score for the current health state. These utility scores were assigned to the patients in the corresponding health states in the model and used to estimate the number of QALYs gained.

### Analysis

The objective of the cost-utility analysis was to evaluate the economic consequences of choosing anastrozole over tamoxifen, in terms of costs, QALYs gained and the cost per QALY gained. The incremental cost per QALY gained was calculated as the difference in cost between anastrozole and tamoxifen divided by the difference in QALYs using a Monte Carlo simulation of 5000 runs (Crystal Ball® 2000). Results were presented as means for costs and effects separately, and as a cost-effectiveness acceptability curve. The latter curve shows the probability that the incremental cost per QALY gained lies below a specified monetary threshold (van Hout *et al*, 1994).

To assess the robustness of the study results with respect to the base-case assumptions, key parameters and assumptions were varied in a sensitivity analysis. In particular, the analysis was carried out probabilistically.

*β-*Distributions are commonly accepted to be used to model transition probabilities (Briggs, 2001). Parameters of the *β* were calculated based on patient numbers in the trial. *γ*-Distributions were assumed to be a good fit for skewed and non-negative total cost data for each item (see Table 2). The overall level of uncertainty in the results was expressed as a cost-effectiveness acceptability curve. The acceptability curve provides an exact fit for the distribution of the incremental cost-effectiveness ratio (ICER) derived from the simulation. The curve shows the probability that the ICER is less than a specified cost per QALY and, therefore, shows the probability that the ratio is cost-effective relative to any chosen ICER threshold. The contribution of the parameters to the variance of the ICER was also estimated.

In addition, a one-way sensitivity analysis was also carried out whereby the parameters were varied individually and expressed as a tornado chart.

The extent to which the uncertainty of parameters was explored in the sensitivity analysis varied by parameter (see Tables 1, 2, 3). ATAC trial data were used as the basis for determining the uncertainty in efficacy parameters. An s.d. equalling 90% of the mean or the variation in responses obtained from the physician survey was used to determine the uncertainly in costs.

The impact of varying the duration of the benefit of anastrozole was also examined. In the base-case analysis it was assumed that the benefits of anastrozole would continue out to 10 years from initiation of treatment. In the sensitivity analysis, two other scenarios were explored. In the first scenario, the incremental benefits of anastrozole were assumed to extend out to lifetime. In the second scenario, it was assumed that the benefits of anastrozole would be limited to the median duration of follow-up of the ATAC trial (i.e. 6 years).

## RESULTS

### Clinical outcomes and costs

**Figure 4:**
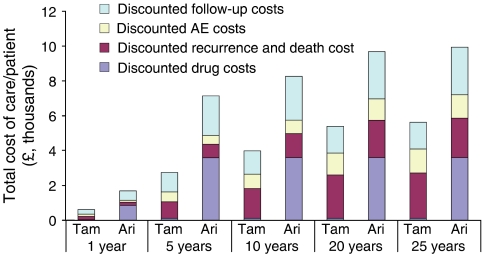
Total cost of care per patient treated with either tamoxifen (Tam) or anastrozole (Ana) over 5, 10, 20 and 25 years. The cost of ‘Drugs’ refer to the acquisition cost of anastrozole and tamoxifen only. The cost of ‘follow-up’, ‘adverse events’, ‘recurrence and palliative care’ include the cost of drug treatment.

When the 5-year ATAC trial results were extrapolated to 25 years, anastrozole and tamoxifen were associated with mean QALYs of 9.21 and 8.96 years, respectively, per patient. Anastrozole was also associated with a longer projected (and discounted) overall mean survival duration at 25 years (9.46 *vs* 9.23 life-years) and a higher estimated (discounted) cumulative mean cost per patient at 25 years (£9935 *vs* £5620), respectively, compared with the tamoxifen group. The per patient results are shown in Table 4. The higher cumulative drug acquisition cost with anastrozole was partly offset by lower costs for treating recurrences and palliative care (Table 4). Consequently, the contribution of the acquisition cost of anastrozole to total costs of care decreased from 50% at 5 years to 36% at 25 years (Figure 4).

### Cost-effectiveness findings

**Figure 5:**
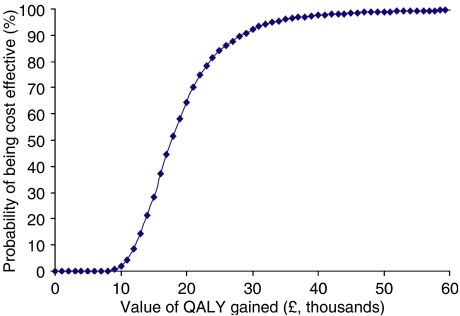
Anastrozole cost-effectiveness acceptability curve for postmenopausal women with hormone receptor-positive early breast cancer (25-year data); QALY, quality-adjusted life-year.

In a model anastrozole was estimated to lead to a gain of 0.244. QALYs (or 0.231 life-years) at an additional cost of £4315 per patient over an actuarial time horizon of 25 years. The results partly arise due to the recurrences avoided with anastrozole compared with tamoxifen and partly due to anastrozole's superior side effect profile compared with tamoxifen. This resulted in an estimated incremental cost-effectiveness of anastrozole compared with tamoxifen of £17 656 per QALY gained. The incremental cost per life-year gained was £18 702. If the time horizon was limited to 5 or 10 years, then the corresponding cost per QALYs would be £219 950 and £47 489, respectively. However, these results assume no difference in costs and no difference in QALYs between anastrozole and tamoxifen beyond these time horizons. A simulation of 5000 runs of the model generated a cost-effectiveness acceptability curve that indicated that there was a greater than 90% probability that the cost per QALY gained with anastrozole would be lower than £30 000 and of the order of 65% that it would be lower than £20 000 (Figure 5). The incremental cost-effectiveness ratio had a 95% non-parametric probability interval (PI) of £10 280 to £39 235 per QALY gained as reflected in the acceptability curve.

### Sensitivity analysis

The overall uncertainty of the ICER is represented by the acceptability curve shown in Figure 3. All the parameters of the model, including the utility values shown in Table 3, were included in the probabilistic sensitivity analysis. Table 5 shows the parameters that had the largest contribution to the variance of the ICER in the probabilistic analysis. The table shows that the ICERs are largely dependent on the parameters of the Weibull regression, the probability of receiving bisphosphonate treatment, the cost of bisphosphonate treatment and the proportion of patients with a distant recurrence if relapsing. The results of the model were robust to changes in key parameters and assumptions in the sensitivity analysis including changes in fracture rates and the parameters of the distributions assumed for costs and probabilities. The regression coefficient of the Weibull time to recurrence curve was the most sensitive parameter in the stochastic analysis (Table 5). Other parameters that contributed to the variation of the ICER were the probability of receiving bisphosphonate and the cost of bisphosphonate treatment, that is, how long patients receive it. Even allowing for the variability in the utility values shown in Table 3, utilities had little impact on the results. If a more pessimistic scenario for utilities was used, then this would improve the results in favour of anastrozole because of the latter's superior efficacy and safety profile *vs* tamoxifen. These corresponded to the results of the one-way sensitivity analysis.

The sensitivity analysis also examined the key base–case assumption that the recurrence curves continued to show benefit for anastrozole out to 10 years from initiation of treatment. When the benefit was limited to the median duration of follow-up of the ATAC trial (i.e. 6 years), the incremental cost-effectiveness ratio of anastrozole *vs* tamoxifen was £23 995 per QALY gained. When the benefit of anastrozole was extended to the lifetime of the patient, this value dropped to £14 441 per QALY gained. A sensitivity analysis was also performed using the alternative discount rates of 6% for costs and 1.5% for benefits as used by NICE in their analysis. When these alternative discount rates were used, the cost per QALY was reduced to £13 185.

## DISCUSSION

The analysis predicts that the benefit of anastrozole *vs* tamoxifen as adjuvant therapy in postmenopausal women with HR+ early breast cancer would translate over an actuarial 25-year time frame into fewer breast cancer deaths and a consequent improvement in survival (231 years gained) and quality of life (244 QALYs gained) among a cohort of 1000 patients. The benefits were provided at an incremental cost of £17 656 per QALY (95% PI: £10 280 to £39 235) gained. This value overlaps the £20 000–£30 000 range NICE has defined as generally acceptable for new technologies (National Institute for Health and Clinical Excellence, 2004). Given the recent recommendations that optimal adjuvant hormonal therapy for a postmenopausal woman with HR+ early breast cancer should include an aromatase inhibitor (such as anastrozole) as initial therapy or after treatment with tamoxifen (Winer *et al*, 2005), such findings will clearly be of relevance to healthcare decision makers both in the United Kingdom and elsewhere.

The study was based on a Markov model for which a number of assumptions were made. Consequently, as with any modelling exercise, there are potential limitations that warrant consideration. For example, the choice of time horizon could potentially influence the results of the model. The study examined a 25-year time horizon. This time frame was chosen because of the need to consider the long-term benefits of treatment and because 25 years would seem to represent a time point when the majority of patients would have died. The incremental cost-effectiveness of £17 656 per QALY gained would be lower over longer time horizons and higher over shorter time horizons. The reason for this relationship is that most of the costs are drug related and occur early on, while the survival effects occur predominantly later on.

The model also assumed that anastrozole provided incremental benefit compared with tamoxifen out to 10 years from the initiation of therapy. This assumption was based on the continuing divergence of the recurrence curves observed during a median of 68 months' follow-up in the ATAC Trialists' Group (2005) and the previous observation that tamoxifen provides a durable benefit (Early Breast Cancer Trialists' Group, 2005). This key assumption did not appear to substantially influence the results. Indeed, when this assumption was explored in the sensitivity analysis, it was found that anastrozole retained an acceptable level of cost-effectiveness (£23 995 per QALY gained) when the benefit of anastrozole was only extended to the end of the median duration of follow-up in the ATAC trial (that is, 6 years). As for the base–case analysis, this value remains within the generally accepted threshold range for cost-effectiveness in the UK (Towse and Pritchard, 2002).

Another potential limitation of the study is that the model relied upon clinical expert opinion to define treatment from which resource consumption was estimated. Despite the importance of breast cancer in the national health policy of the United Kingdom, there are few published data regarding the costs of treating breast cancer. This is due to the lack of large administrative databases linked with diagnosis that would provide the basis for analysis of resource use, rendering most cost-effectiveness studies to rely on published sources (Karnon and Brown, 2002). The inclusion of real-life data from retrospective data sets or prospective naturalistic studies concerning adjuvant hormonal therapy in the United Kingdom would therefore strengthen the model.

Another notable finding of the present study was that the cost-effectiveness of anastrozole was not sensitive to differences in adverse event rates, including the risk and associated costs of hip fracture. This is despite the model making allowances for some patients to receive prophylactic therapy during treatment with anastrozole, and an assumption that the risk of hip fracture extended for an additional 3 years beyond completion of therapy. The fact that such allowances had little effect on the study findings strengthens the stability of the model.

Two other studies have been carried out to evaluate the cost-effectiveness of anastrozole *vs* tamoxifen in the primary adjuvant breast cancer setting (Hillner, 2004; Locker, 2004). In the Locker study, the incremental cost-effectiveness for anastrozole *vs* tamoxifen was $23 740 per QALY gained, a value that lies within the threshold range that is generally accepted for reimbursement in the USA ($50 000–$100 000 per QALY gained). In the Hillner study, the incremental cost-effectiveness of anastrozole was $75 900 per QALY gained. However, neither of the findings is readily comparable to the present investigation due to differences in perspective (i.e. United States *vs* United Kingdom). Furthermore, while the Locker study has many similarities to the present study with regards to model design, the Hillner study differed in many respects such as in model structure, time horizon, healthcare resource utilisation and study assumptions. For example, while both the present analysis and the Locker study used the most recent ATAC clinical trial data (5-year update) modelled over 25 years, the Hillner study used the 4-year update modelled over a 20-year time horizon. Moreover, Hillner's model assumed an odds ratio of 1.6 for hip fractures during anastrozole therapy, which is higher than that seen in the completed treatment analysis of the ATAC trial (1.2 (95% confidence interval, 0.74–1.93); *P*=0.5) (ATAC Trialists' Group, 2005). The exact hip fracture odds ratio was used in both the present study and the Locker model. At no increased risk of hip fracture, the incremental cost-effectiveness of anastrozole in Hillner's model was $25 300 per QALY gained, which is comparable with the Locker result. While bearing in mind the limitations of any direct comparison of the present study with the above study, this equates to an incremental cost of £15 150 per QALY gained (exchange rate £1=$1.67, October 2005), which is similar to that found in the present study (£17 656 per QALY gained), falling within NICE's generally accepted threshold range for cost-effectiveness in the United Kingdom. Also, in the present study, the results were robust to changes in the odds ratio for hip fractures, with only a small increase in cost/QALY to £19 077 if the odds ratio increased to 1.6. This is largely due to the relatively low incidence of hip fractures in both treatment arms and the assumption of a low level of bisphosphonate usage in patients receiving anastrozole in the United Kingdom.

Cost-effectiveness ratios are recognised by NICE as an important aid to decision making. NICE also has a strong preference for expressing health gains in terms of QALYs. However, the NICE Appraisal Committee does not use a fixed threshold for incremental cost-effectiveness ratios above which a technology would automatically be defined as not cost-effective or below which it would. On the other hand, comparisons with the incremental cost-effectiveness ratios for previous treatments recommended by NICE suggest that there is a high probability that the incremental cost-effectiveness ratio for anastrozole relative to tamoxifen would fall within the range that is likely to be acceptable (i.e. <£20 000–£30 000 per QALY gained) (Towse and Pritchard, 2002; National Institute for Health and Clinical Excellence, 2004). This substantiates that anastrozole represents acceptable value for money, relative to tamoxifen, in the treatment of postmenopausal women with HR+ early breast cancer. Moreover, the cost-effectiveness of anastrozole compares favourably with other cancer therapies recommended by NICE and commonly used by the NHS, such as trastuzumab combination therapy for treatment of advanced breast cancer (National Institute for Health and Clinical Excellence, 2002) and imatinib for treatment of chronic myeloid leukaemia (National Institute for Health and Clinical Excellence, 2003). A 2003 budget impact study estimated that use of anastrozole would cost an additional £19.3 million over 3 years, based on projected uptake of anastrozole over tamoxifen of 17, 34 and 50% in each of the following 3 years, respectively (Benedict and Christie, 2003). By comparison a NICE assessment estimated that the treatment of all women with osteoporosis would cost £0.9–1.5 billion over 5 years using bisphosphonates (alendronate, etidronate, risedronate) and raloxifene, increasing to more than £3.0 billion if teriparatide is included (National Institute for Health and Clinical Excellence, 2005).

This study indicates whether reimbursement is warranted given the existing evidence. However, the study does not assess whether the existing evidence is sufficient for making those kinds of decisions or whether additional research would be beneficial. (Sculpher and Claxton, 2005). A formal value of information analysis is outside the scope of this study. On the other hand, given that the greatest source of uncertainty is the long-term extrapolation of recurrence and mortality rates, it would seem appropriate for the model to be updated as longer-term follow-up data from ATAC becomes available.

## CONCLUSIONS

The results of this study demonstrate that anastrozole is a cost-effective alternative to tamoxifen (i.e. £17 656 per QALY gained) for the adjuvant treatment of postmenopausal women with HR+ early breast cancer from the UK NHS perspective when compared with NICE's thresholds of £20 000–£30 000 per QALY. In particular, the discounted incremental cost per QALY gained compared favourably with other commonly accepted cancer treatments. These results provide economic support for the recent ASCO recommendations that optimal therapy for these women should now include use of an aromatase inhibitor to lower the risk of breast cancer recurrence (Winer *et al*, 2005). Anastrozole can therefore be considered to be an effective and well-tolerated therapy that provides value for money in the adjuvant treatment of postmenopausal women with HR+ early breast cancer in the UK.

## Figures and Tables

**Table 1:** Outcome probabilities used in the model

	**Anastrozole**	**Tamoxifen**	**Distribution**	**Probabilistic parameters**
Distant recurrences as a proportion of all recurrences during recurrence benefit^a^	0.66	0.60	*β*	Anastrozole: *α*, 186; *β*, 96
Tamoxifen: *α*, 222, *β*, 148				
				
*Adverse events* b				
Life-threatening	0.047	0.066	*β*	Anastrozole: *α*, 142; *β*, 2950
Tamoxifen: *α*, 201, *β*, 2893				
Non life-threatening	0.698	0.657	*β*	Anastrozole: *α*, 2163; *β*, 929
				
Tamoxifen: α, 2037, *β*, 1057				
None	0.255	0.277	Remainder	
				
*Following local/regional recurrence*				
Distant metastases-free at 5 years^c^		0.52	*β*	*α*, 73; *β*, 67
Distant metastases-free after 5 years^d^		0.77	*β*	*α*, 91; *β*, 27
Death due to breast cancer^e^		0.222	*β*	*α*, 26; *β*, 140
				
*Following distant recurrence*				
Overall survival at 2 years^f^		0.50	*β*	*α*, 155; *β*, 155

a Note that the overall number of recurrences was lower in the anastrozole arm (282) than in the tamoxifen arm (370); percentages are calculated based on recurrences occurring as a first event, estimated from ATAC data (anastrozole 186, tamoxifen 222), and the uncertainty in these estimates was compensated for by assigning a distribution to these estimates in the probabilistic sensitivity analysis; the estimates assume the benefits of anastrozole continued out to 10 years from initiation of treatment.

b From the 68-month median follow-up of ATAC trial patients (ATAC Trialists' Group, 2005).

c From Kamby and Sengeløv (1997).

d From Moran and Haffty (2002).

e Estimated from the median 68-month follow-up data of ATAC trial patients (ATAC Trialists' Group, 2005).

f From Stockler *et al* (2000).

**Table 2:** Estimated costs of medical management, death and adverse events used in the model^a^

	**Cost (£)**	**s.d.^b^**
*Treatment/diagnosis (cost/event)*		
At treatment initiation	90	38
Diagnosis of recurrence	808	92
Treatment for local/regional recurrence	2606	2085^a^
Treatment for distant recurrence	3563	2850^a^
		
*Follow-up and monitoring (cost/cycle)*		
Follow-up for local/regional recurrence	143	66
Follow-up for distant recurrence	199	95
		
*Routine follow-up*		
Years 1	70	20
Years 2+	43	17
Follow-up off treatment due to remission	24	18
Follow-up off treatment due to adverse events	51	43
		
*Death (cost/year)*		
Death from breast cancer^c^	3783	3404
Death from other causes	500	450
		
*Adverse event*		
Fractures^d^		NA
Wrist	1463	NA
Spine	2915	NA
Hip	10 682	NA
Ischaemic cerebrovascular event	6299	NA
Deep venous thromboembolism^e^	2110	NA
Endometrial cancer	2245	NA
Hysterectomy	1873	NA
Ischaemic cardiovascular disease	3251	NA
Vaginal bleeding	1407	NA
Hot flushes	239	NA
Musculoskeletal disorders	533	NA
Mood disturbances	109	NA
Fatigue	20	NA
Nausea and vomiting	20	NA
Vaginal discharge	240	NA
Bisphosphonate treatment	1432	NA

Abbreviation: NA=not applicable.

a On the basis of physician survey, MEDTAP unit cost database and NHS reference costs (Department of Health, 2003), except where stated.

b Costs were assumed to follow a *γ*-distribution.

c From Coyle *et al* (1999).

d From Iglesias *et al* (2002).

e From Gordois *et al* (2003).

**Table 3:** Deterministic mean utility scores used in the model (*n*=23)

		**Probabilistic parameters**	
**Utility item**	**Mean (s.d.)**	**α**	**β**
Disease-free state, no adverse events	0.989 (0.010)	106.60	1.19
Common adverse events (tamoxifen)	0.970 (0.041)	15.82	0.49
Common adverse events (anastrozole)	0.962 (0.055)	10.66	0.42
Vaginal bleeding	0.933 (0.099)	5.02	0.36
Endometrial cancer	0.913 (0.101)	6.20	0.59
Wrist fracture	0.916 (0.099)	6.28	0.58
New contralateral breast cancer	0.914 (0.097)	6.72	0.63
Local/regional recurrence	0.911 (0.098)	6.78	0.66
Deep vein thromboembolism	0.922 (0.107)	4.87	0.41
Pulmonary embolism	0.890 (0.166)	2.27	0.28
Spinal fracture	0.894 (0.189)	1.48	0.18
Hip fracture	0.858 (0.199)	1.78	0.29
Hormonal therapy for distant recurrence	0.882 (0.105)	7.44	1.00
Chemotherapy for distant recurrence	0.710 (0.254)	1.56	0.64
Current health	0.933 (0.069)	11.32	0.81
Hysterectomy	0.899 (0.101)	7.10	0.80

**Table 4:** Outcomes and cost of care per patient (discounted 25-year data)

	**Outcomes**		**Cost (£)**				
**Treatment**	**QALYs (years)**	**LYG (years)**	**Drug**	**Adverse events**	**Follow-up**	**Recurrence and palliative care**	**Total**
Anastrozole	9.206	9.464	3598	1347	2735	2254	9935
Tamoxifen	8.962	9.234	113	1366	1539	2603	5620

Abbreviations: LYG=life-years gained; QALYs=quality-adjusted life-years gained.

**Table 5:** Sensitivity of the incremental cost/QALY ratio to parameters

**Parameter**	**Contribution of parameters to the variability of the ICER (%)**	**Rank correlation**
Weibull regression parameter (*α* coefficient)^a^	63.0	−0.85
Weibull regression parameter (constant)^a^	14.2	0.40
Cost of bisphosphonate treatment	5.5	0.25
Probability of receiving bisphosphonate treatment	3.3	0.19
Proportion of patients with a distant recurrence if relapsing	1.5	0.13
Utility of chemotherapy for distant cancer	1.9	0.15

Abbreviations: ICER=incremental cost effectiveness ratio; QALY=quality-adjusted life year.

a Correlated parameter.
